# First-in-human phase I clinical trial of RG7356, an anti-CD44 humanized antibody, in patients with advanced, CD44-expressing solid tumors

**DOI:** 10.18632/oncotarget.11098

**Published:** 2016-08-05

**Authors:** C. Willemien Menke-van der Houven van Oordt, Carlos Gomez-Roca, Carla van Herpen, Andrew L. Coveler, Devalingam Mahalingam, Henk M. W. Verheul, Winette T. A. van der Graaf, Randolph Christen, Dominik Rüttinger, Stefan Weigand, Michael A. Cannarile, Florian Heil, Michael Brewster, Antje-Christine Walz, Tapan K. Nayak, Ernesto Guarin, Valerie Meresse, Christophe Le Tourneau

**Affiliations:** ^1^ Department of Medical Oncology, VU University Medical Center, Amsterdam, The Netherlands; ^2^ Clinical Research Unit, Department of Medical Oncology, Institut Claudius Regaud, Institut Universitaire du Cancer de Toulouse – Oncopole, Toulouse, France; ^3^ Radboud University Medical Center, Nijmegen, The Netherlands; ^4^ Department of Medicine, Division of Oncology, University of Washington, Seattle, WA, USA; ^5^ Cancer Therapy and Research Center, University of Texas Health Science Center, San Antonio, TX, USA; ^6^ Product Development, Safety Risk Management, Roche, Basel, Switzerland; ^7^ Pharma Research & Early Development, Roche Innovation Center, Penzberg, Germany; ^8^ Pharma Research & Early Development, Roche Innovation Centre, Welwyn, UK; ^9^ Pharma Research & Early Development, Roche Innovation Center Basel, Basel, Switzerland; ^10^ Department of Medical Oncology, Institut Curie, Saint-Cloud & Paris, France, and Versailles-Saint-Quentin-en-Yvelines University, Versailles, France

**Keywords:** RG7356, anti-CD44 humanized antibody, advanced solid tumors, advanced CD44-expressing solid malignancies, phase I trial

## Abstract

Transmembrane glycoprotein CD44 is overexpressed in various malignancies. Interactions between CD44 and hyaluronic acid are associated with poor prognosis, making CD44 an attractive therapeutic target. We report results from a first-in-human phase I trial of RG7356, a recombinant anti-CD44 immunoglobulin G1 humanized monoclonal antibody, in patients with advanced CD44-expressing solid malignancies.

Sixty-five heavily pretreated patients not amenable to standard therapy were enrolled and received RG7356 intravenously biweekly (q2w) or weekly (qw) in escalating doses from 100 mg to 2,250 mg. RG7356 was well tolerated. Most frequent adverse events were fever, headache and fatigue. Dose-limiting toxicities included headache (1,500 mg q2w and 1,350 mg qw) and febrile neutropenia (2,250 mg q2w). The maximum tolerated dose with q2w dosing was 1,500 mg, but was not defined for qw dosing due to early study termination. Clinical efficacy was modest; 13/61 patients (21%) experienced disease stabilization lasting a median of 12 (range, 6–35) weeks. No apparent dose- or dose schedule-dependent changes in biological activity were reported from blood or tissue analyses. Tumor-targeting by positron emission tomography (PET) using ^89^Zr-labeled RG7356 was observed for doses ≥200 mg (q2w) warranting further investigation of this agent in combination regimens.

## INTRODUCTION

CD44 is a single-chain, transmembrane glycoprotein involved in cell-cell and cell-matrix interactions [1]. Interaction between CD44 and its main ligand, hyaluronic acid (HA), may be important in tumor growth and treatment resistance via cell proliferation, differentiation, and migration [1]. These interactions further influence interstitial fluid pressure, tumor-associated macrophage attraction, and angiogenesis [1, 2]. CD44 encompasses a diverse family of molecules originating from alternative splicing and posttranslational modifications in different cells [1, 2]. CD44 standard (CD44s) and variant (CD44v) isoforms are aberrantly expressed in various cancers [3] and their interaction with HA influences cell migration and homing [4]. CD44, also expressed on cancer stem cells (CSC) in various tumors, is associated with chemoresistance and tumor regrowth following standard therapy [5]. As CD44 overexpression is linked to tumor aggressiveness and metastatic potential in many tumors, it is an attractive therapeutic target [1, 3]. The anti-CD44 antibody bivatuzumab, directed against the variable region CD44v6, has demonstrated clinical activity in phase I studies; however, lethal toxic epidermal necrolysis halted further development [6–8].

Investigational RG7356, a novel recombinant immunoglobulin G1 humanized monoclonal antibody (mAb), selectively binds near the HA-binding region of the extracellular domain of all CD44 isoforms. Preclinical studies suggest that higher levels of HA and expression of CD44s *versus* CD44v on cells are important for RG7356 activity [4]. RG7356 has also demonstrated growth inhibition of several CD44-expressing tumor xenografts (Roche internal data). Its mode of action has been postulated to include phagocytosis of CD44^−^ positive cancer (stem) cells, which in preclinical studies has been suggested to involve Fc-mediated activation of macrophages [4], as well as being involved in direct cell killing of CD44^−^ positive cancer cells.

We report a first-in-human, multicenter, phase I clinical trial of RG7356 in patients with metastatic or locally advanced CD44-expressing solid malignancies not amenable to standard therapy. Biodistribution of RG7356 was evaluated using ^89^Zr-RG7356 positron emission tomography (PET).

## RESULTS

### Patient characteristics

Sixty-five patients were enrolled consecutively from June 2011 through November 2013. In Arm A, 40 patients received RG7356 biweekly (q2w) in 8 dose cohorts (100 to 2,250 mg), and 12 patients received the weekly (qw) regimen (675-mg and 1,350-mg cohorts). Thirteen patients in the substudy imaging group (Arm B) received 1 mg ^89^Zr-RG7356 after 0-mg to 674-mg unlabeled RG7356 prior to PET imaging (Supplemental Material, online only). Patients received a median of 3.5 prior therapies in Arm A and 3.0 in Arm B (Table 1).

**Table 1:** Patient characteristics

Characteristic	Arm A		Arm B
	q2w *N* = 40	qw *N* = 12	q2w *N* = 13
Median age (range), years	65 (41–80)	57 (25–65)	61 (40–79)
Male, n (%)	22 (55)	5 (42)	10 (77)
Race, n (%)			
Asian	2 (5)	0	1 (8)
Black/African American	2 (5)	2 (17)	0
White	35 (88)	10 (83)	12 (92)
Other	1 (3)	0	0
ECOG, *n* (%)			
0	11 (28)	5 (42)	2 (15)
1	27 (68)	6 (50)	10 (77)
2	2 (5)	1 (8)	1 (8)
Primary cancer, *n* (%)			
Colon/large intestine	15 (38)	0	4 (31)
Rectum	8 (20)	0	1 (8)
Breast	2 (5)	3 (25)	0
Melanoma	3 (8)	2 (17)	1 (8)
Head and neck	2 (5)	0	2 (15)
Skin	1 (3)	1 (8)	0
Soft tissue	1 (3)	1 (8)	0
Uterus	1 (3)	1 (8)	0
Cervix	1 (3)	0	2 (15)
Esophagus, gastric, gastroesophageal junction	3 (7)	0	1 (8)
Kidney	0	1 (8)	0
Pancreas	0	1 (8)	0
Thymus	1 (3)	0	0
Other^a^	2 (3)	1 (8)	2 (15)
Median line of prior therapy (range)	3.5 (0–9)	3.5 (0–7)	3.0 (1–7)

Abbreviations: ECOG, Eastern Cooperative Oncology Group; qw, weekly; q2w, biweekly

a “Other” includes bone, adenoid cystic carcinoma of glandula submandibularis, cholangiocarcinoma, chondrocarcinoma, ear, nasopharynx, and eye.

### Safety and tolerability

Overall, 317 treatment-related adverse events (AEs), mostly mild to moderate, were reported in 61 patients, with comparable event rates in arms A and B (Table 2). Grade 3 and 4 AEs were reported in 25% (16/65) and 5% (3/65) of patients, respectively. Most common treatment-related AEs included headache (38/65, 58%) and pyrexia (30/65, 46%). Infusion-related reactions (IRRs) did not appear to be dose schedule-dependent and were predominantly observed during the first infusion. Most IRRs were grade 1 or 2, well managed with recommended premedication, and resolved without clinical sequelae. Overall, 52 serious AEs were reported in 31 patients; 11 events (pyrexia, headache, abdominal pain, febrile neutropenia, and nausea) were considered study drug related.

**Table 2:** Safety overview

Category	Arm A		Arm B	Total *N* = 65	
	q2w *N* = 40	Weekly *N* = 12	q2w *N* = 13		
Any AE, *n* (%)	40 (100)	12 (100)	13 (100)	65 (100)	
Total number of AEs	378	128	113	619	
Related AE, *n* (%)	39 (98)	10 (83)	12 (92)	61 (94)	
Total number of related AEs	218	57	42	317	
Related grade ≥3 AE, *n* (%)	12 (30)	3 (25)	2 (15)	17 (26)	
Total number of related grade ≥3 AEs	14	5	3	22	
SAE, *n* (%)	16 (40)	8 (67)	7 (54)	31 (48)	
Total number of SAEs	24	14	14	52	
Related SAE, *n* (%)	5 (12)	1 (8)	3 (23)	9 (14)	
Total number of related SAEs	5 (12)	1 (8)	5 (38)	11 (17)	
Infusion-related reactions, *n* (%)	27 (68)	7 (58)	10 (77)	44 (68)	
Total number of infusion-related reaction events	72	15	27	114	
Serious infusion-related reactions *n* (%)	2 (5)	0	0	2 (3)	
AE leading to withdrawal, *n* (%)	2 (5)	2 (17)	2 (15)	6 (9)	
Deaths, *n* (%)	16 (40)	2 (17)	6 (46)	24 (37)	
DLT^a^	2 (5)	1 (8)	0	3 (5)	
**Treatment-related**^b^ **AEs in ≥10% of patient population, n (%)**^c^	39 (75)		12 (92)	**Total**	
				51 (78)	**Proportion of treatment-related AEs grade ≥ 3**^d^
Headache	26 (65)	6 (50)	6 (46)	38 (58)	2 (4)
Asthenia/fatigue	22 (55)	8 (50)	0	30 (46)	1 (2)
Pyrexia	18 (45)	3 (25)	9 (69)	30 (46)	1 (2)
Chills	10 (25)	1 (8)	4 (31)	15 (23)	0
Nausea	8 (20)	2 (17)	5 (38)	15 (23)	1 (2)
Decreased appetite	9 (23)	3 (25)	2 (15)	14 (22)	1 (2)
Vomiting	6 (15)	3 (25)	3 (23)	12 (18)	1 (2)
Rash/maculopapular rash	5 (13)	4 (33)	0	9 (14)	0
Diarrhea	5 (13)	2 (17)	1 (8)	8 (12)	0
Conjunctivitis	4 (10)	1 (8)	2 (15)	7 (11)	0
Dizziness	7 (18)	0	0	7 (11)	0

Abbreviations: AE, adverse event; DLT, dose-limiting toxicity; q2w, biweekly; qw, weekly; SAE, serious adverse event.

Erythema and dry eye were reported as treatment-related AEs in 4 (6%) and 3 (5%) patients, respectively.

a DLTs occurred in the 1,500 and 2,250 mg q2w cohorts and 1,350 mg qw cohort.

b Investigator assessed.

c Calculated as proportion of total number of patients who experienced any AE in that treatment group.

d Calculated as proportion of total number of patients who experienced any treatment-related AE.

Three dose-limiting toxicities (DLTs) included 2 cases of grade 3 headache (1,500 mg q2w, 1,350 mg qw) and 1 grade 4 febrile neutropenia (2,250 mg q2w). As the first DLT occurred with 1,500 mg q2w, this cohort was expanded to 6 patients; no additional DLT was observed. In the subsequent 2,250 mg q2w cohort, 1 DLT occurred in 3 evaluable patients. The safety study management team decided not to further expand this cohort, as drug exposure was judged insufficient owing to rapid antibody clearance. Instead more frequent (weekly) dosing was implemented to increase drug exposure.

The safety profile of the qw regimen was comparable with that for q2w (Table 2). Lower rates of IRRs (58%) compared with q2w dosing coincided with the introduction of a slower infusion protocol. Thus, the maximum tolerated dose (MTD) for the q2W regimen was determined at 1,500 mg and was not reached in the qw regimen owing to study termination (sponsor decision) prior to any further dose escalation after the highest tested 1,350-mg dose.

In the q2w cohort, interference with the Coombs test post infusion was observed. Subsequent analysis of the qw cohort showed that the indirect and direct Coombs test became positive after the first infusion in 57% (4/7) and 55% (6/11) of patients, respectively, and became negative again in 2 (at 675 and 1,350 mg qw) and 3 patients (at 675, 1,350, and 1,350 mg qw), respectively, at the cycle 1 day 8 assessment (data not shown). No hemolysis was reported. A de-risking strategy was implemented (pre-dose Coombs testing on day 1 of cycles 1 and 8 and at end of treatment visit). No AEs due to this interference have been observed.

Patient withdrawals were due to death (*n* = 1), withdrawal of consent (*n* = 3), and AEs (*n* = 6), which included 3 patients with DLTs. All other withdrawals were due to progressive disease (*n* = 55). None of the 24 deaths in the study were considered study drug related, as assessed by the investigator; all deaths were assessed by the investigators to be due to disease progression.

### Pharmacokinetic assessments

**Figure 1:**
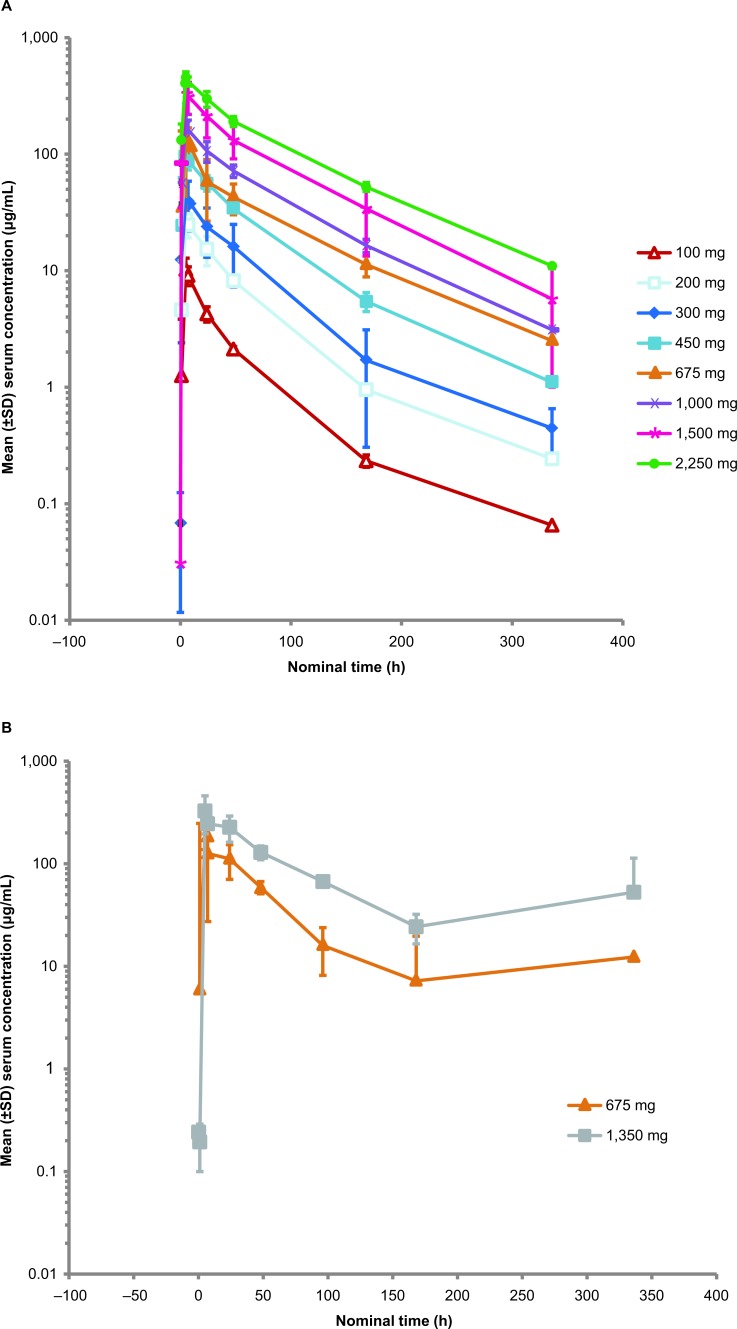
RG7356 serum concentration *versus* time plot in patients in Arm A who received the biweekly (q2w) (A) and weekly (qw) (B) dosing schedules

In Arm A, the mean peak concentration and area under the curve (AUC) at RG7356 doses up to 2,250 mg showed nonlinear pharmacokinetics (PK). More than dose-proportional increases in drug exposure were observed at doses of 100-450 mg, with dose-proportional increases indicative of clearance being a dose-dependent and potentially saturable process that plateaus at higher doses (Figure 1A). Volume of distribution was high and apparent mean half-life of approximately 50-70 hours remained similar across doses. Doubling of exposure was achieved by changing from q2w to qw dosing (Figure 1B).

### Antitumor activity

**Figure 2:**
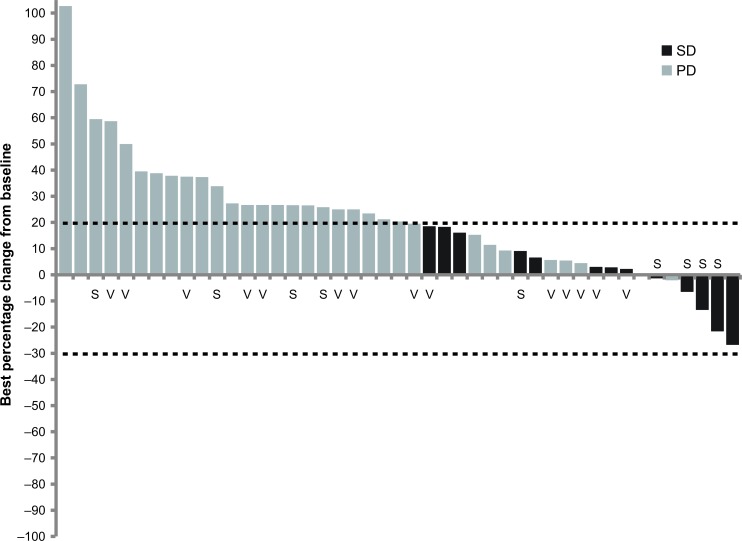
Waterfall plot showing best response of individual patients according to RECIST criteria 45 patients had tumor lesion evaluation during the study. Abbreviations: PD (progressive disease); SD (stable disease).

Following treatment with RG7356, most patients progressed after cycle 2. Median time on treatment was 4 (range, 0.14-34) weeks. Of 61 evaluable patients, 21% (13/61) had stable disease (SD) as best response (Figure 2); 3 patients with colorectal cancer and 1 patient each with thymus and skin cancer showed some tumor shrinkage, although not sufficient to qualify as an objective response according to RECIST criteria. Tissue analysis revealed no specific trend in immune cell infiltration or proliferation (data not shown).

### Biomarker analysis

**Figure 3:**
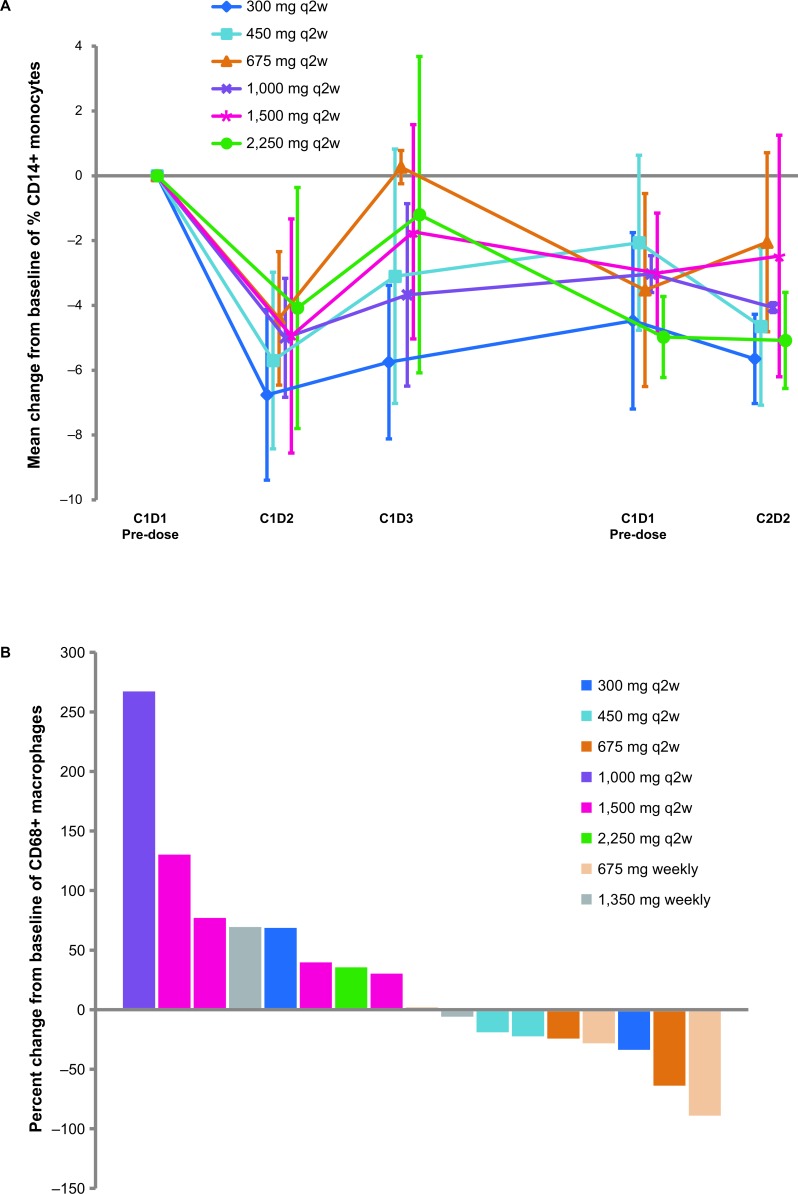

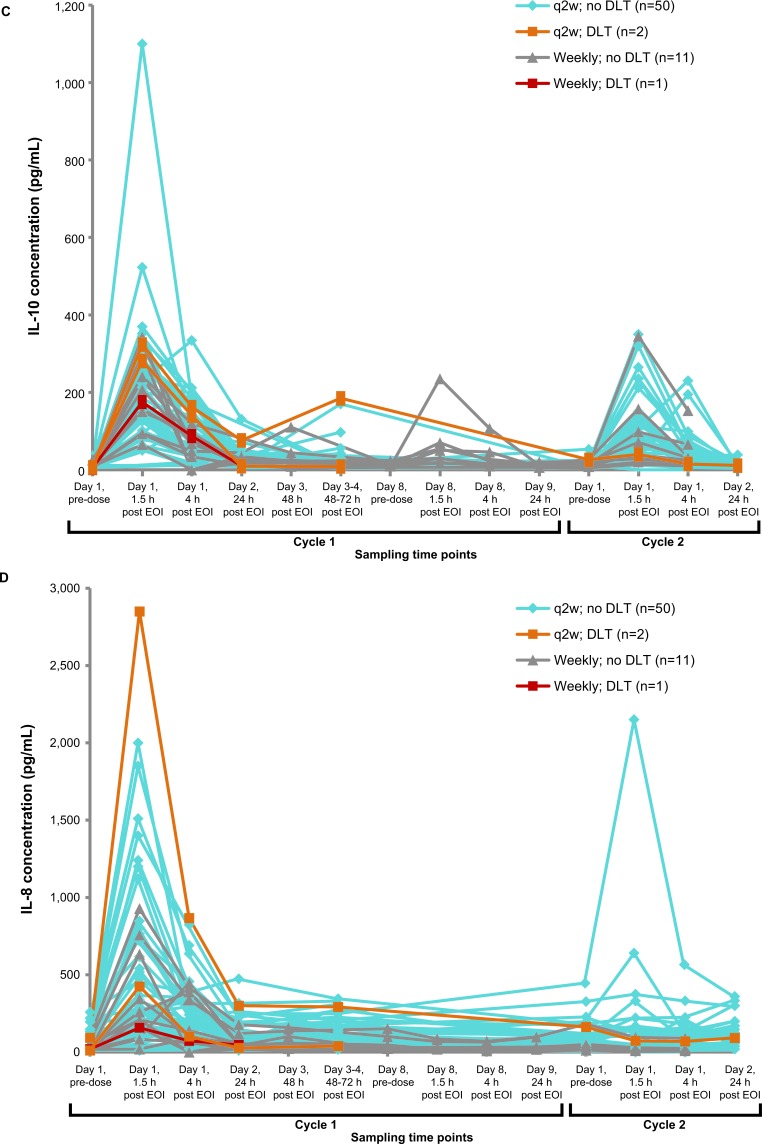

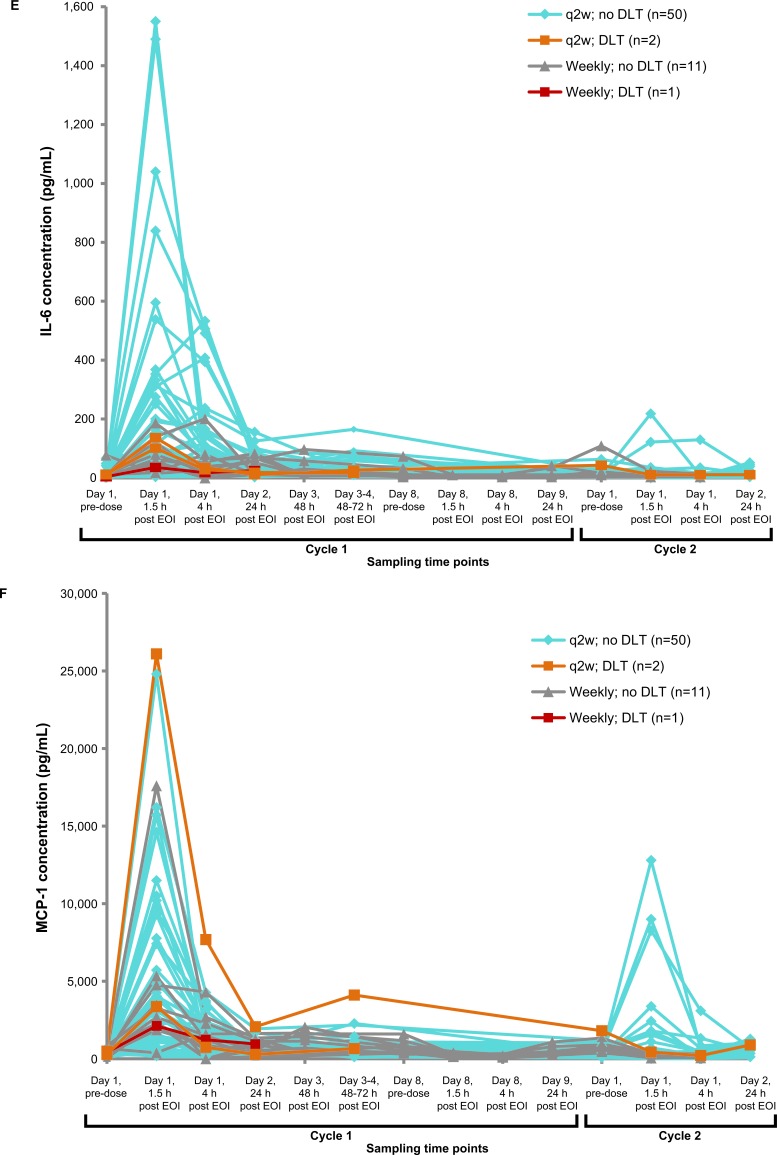
RG7356 induces a temporary reduction from baseline of CD14^+^ peripheral blood monocytes after first infusion (A), does not seem to have an effect on macrophage tumor infiltration (B), and generates a temporary release in cytokines. The latter seems to be independent of dose and/or dose-schedules (C-F). Dose-limiting toxicities (DLTs) occurred in 3 patients during the study: grade 3 febrile neutropenia on study day 18 and 2 cases of headache on study days 4 and 8 (C-F). Abbreviations: C (cycle); D (day); EOI (end of infusion); IL (interleukin); MCP-1 (monocyte chemoattractant protein-1); q2w (biweekly). **A.** Plot of mean changes from baseline of percent of CD14+ (± standard deviation) at RG7356 doses ≥300 mg for the q2w schedule. **B.** Waterfall plot of percent change in CD68+ macrophages. **C.**-**F.** Individual cytokine profiles.

Analysis of peripheral blood cells showed a temporary dose- and dosing regimen-independent decrease of CD14^+^ monocytes after first infusion (Figure 3A). No trend for RG7356-induced CD68^+^ macrophage alteration was observed (Figure 3B). Additionally, no relevant changes in tumor proliferation [Ki67 immunohistochemistry (IHC)], CD44 expression (CD44 IHC), and CD44-mediated signaling (phosphorylated ERK IHC) was demonstrated in tumor tissue with RG7356 treatment (data not shown).

Cytokine release was temporary, peaking at 1.5 hours after the first infusion and returning towards baseline levels within 24 hours after infusion (Figure 3C-3F). Additionally, a secondary cytokine release was observed in some patients upon repeated RG7356 infusion with similar kinetics as the first infusion-related cytokine release. This secondary cytokine release was not correlated to AEs, DLTs, or other biomarkers measured. Overall, cytokine changes do not seem to be dependent on dose level or dose schedule (Figure 3C-3F).

We also investigated whether observed DLTs (Table 2) could be linked to cytokine release in the serum of RG7356-treated patients. The patient who experienced dose-limiting grade 3 febrile neutropenia on study day 18 (Patient 2010) showed the highest temporary increase of monocyte chemotactic protein 1 and inflammatory cytokine interleukin (IL)-8 after 1.5 hours post first infusion compared with remaining patients. However, the pattern of increase for IL-6 and IL-10 was similar compared with the other patients (Figure 3). At the time of the febrile neutropenia event, cytokine levels were not assessed. Two patients experienced dose-limiting headache. Overall, cytokine levels in these 2 patients were similar to those in other patients treated with RG7356.

### Imaging

Early response evaluation with fluorodeoxyglucose (FDG)-PET showed a partial metabolic response according to European Organisation for Research and Treatment of Cancer criteria in 23.5% (8/34) of patients (119 tumor lesions). No correlation between changes in maximum standardized uptake value or metabolic tumor volume and dose exposition was found. Assessment of baseline and on-treatment vascular characteristics in 20 evaluable patients using dynamic contrast-enhanced magnetic resonance imaging (DCE-MRI) showed changes in initial AUC defined over the first 60 seconds post-enhancement, reflecting a mixed pattern of increase and decrease in vascular characteristics and changes in fluid pressure (data not shown). No correlation with clinical efficacy was observed.

**Figure 4:**
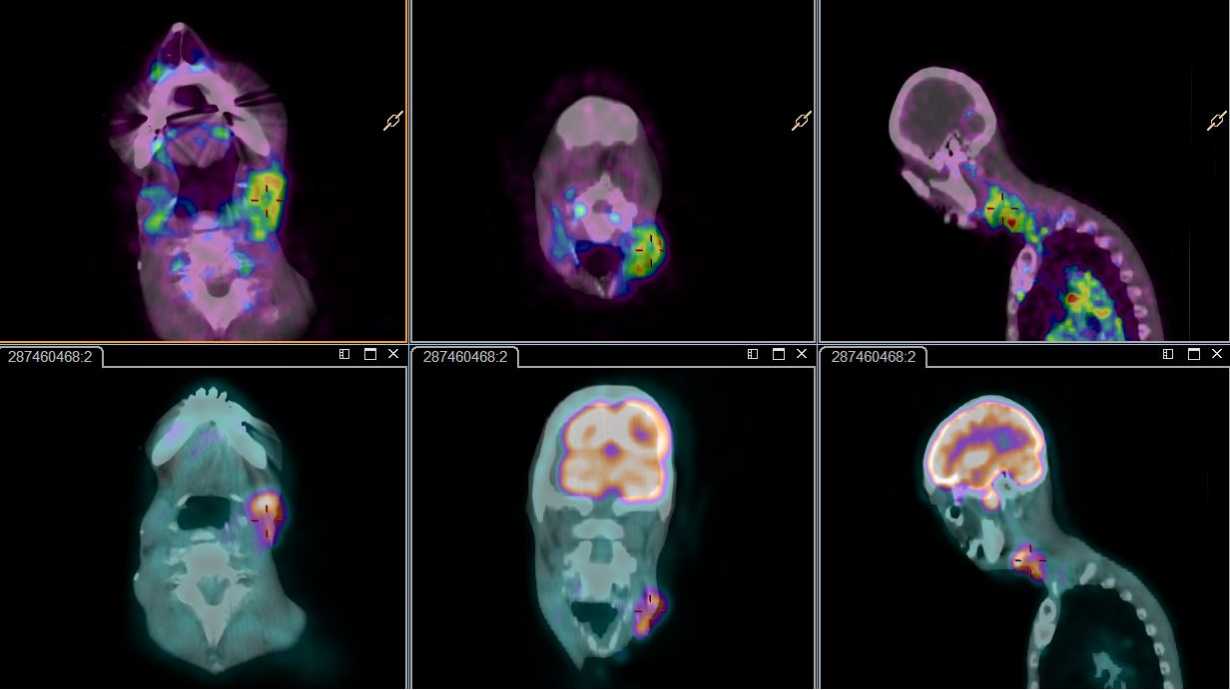
^89^Zr-RG7356 uptake in tumor lesions Representative images of lymph node metastases in left upper neck of a patient with advanced CD44-positive head and neck cancer. *Upper row*: ^89^Zr-RG7356 PET images acquired 5 days post injection. From left to right: axial, coronal and sagittal slices (8 mm) PET images fused with low-dose CT. Enhanced tracer uptake in malignant lymph nodes is shown (indicated by red center cross, SUV_max_ 3.9). *Lower row*: ^18^F-FDG PET images fused with low-dose CT (similar orientation as ^89^Zr-PET; SUV_max_ 7.8). Note that spatial distribution of ^18^F-FDG and ^89^Zr-RG7356 within the lesion is similar with central hypoactivity corresponding to necrosis.

Immuno-PET analysis revealed that ^89^Zr-RG7356 localized to bone marrow, liver, and spleen. Tumor targeting was detected with coadministration of ≥199 mg unlabeled antibody in 7/10 patients (Figure 4). Most lesions seen with FDG-PET also could be detected with ^89^Zr-RG7356 (23/28 lesions; data not shown).

## DISCUSSION

This first-in-human phase I clinical trial with RG7356, a mAb targeted to the constant region of CD44, showed an acceptable safety profile in patients with advanced, CD44-expressing solid tumors. The study was terminated early due to no evidence of a clinical and/or pharmacodynamic (PD) dose-response relationship with RG7356, but not due to safety concerns. Consequently, the optimal biological dose schedule was not achieved. Headache and pyrexia were the most common AEs, the majority of which was related to IRRs most frequently occurring during the first infusion cycle. Two cases of grade 3 headache events occurred at the highest doses. Prior preclinical analysis had shown some accumulation of mononuclear cells in the meninges of cytomologous monkeys with RG7356 treatment (data on file) and aseptic meningitis occurred in 2 patients with acute myeloid leukemia (AML) treated with RG7356 (Vey et al, Phase I clinical study of RG7356, an anti-CD44 humanized antibody, in patients with relapsed/refractory acute myeloid leukemia; submitted to Oncotarget), possibly due to an inflammatory response in the meninges. Exploratory analyses showed no relationship between drug exposure, infusion-related cytokine release, and other AEs. Additionally, no accumulation of ^89^Zr RG7356 to the meninges has been observed.

Bivatuzumab-mertansine, an antibody against CD44v6 linked to the anti-tubulin agent mertansine, was previously associated with significant skin toxicity, including a case of lethal epidermal necrolysis [6]. The efficient targeting of mertansine, which is highly toxic to skin, was most likely responsible for this toxicity. Despite abundant expression of CD44 in skin tissue of patients included in the present study (data not shown), RG7356 appeared safe and associated AEs were predominantly grade 1.

Clinical efficacy of RG7356 was modest with SD observed at 8 weeks in 21% of patients. Analysis of potential biomarkers showed reduction of CD14+ monocytes in peripheral blood suggesting a PD effect of RG7356; one of the proposed modes of action is to trigger direct antitumor effects by activating CD68^+^ macrophages via Fc/FcgR interaction to phagocytose CD44-positive tumor cells. Reduction of circulating CD14^+^ monocytes might anticipate a migration and subsequent differentiation of monocytes into tumor tissue. However, no significant increase in CD68^+^ macrophages in tumor tissue was observed under RG7356 treatment.

Plasma half-life was shorter than expected and, although qw dosing doubled RG7356 exposure, there was no improvement in antitumor activity. Deamidation of asparaginases in the complementarity determining region of intact antibody in plasma was demonstrated, whereby RG7356 is converted to a binding-impaired molecule that remains in circulation (data not shown). Thus, antibody levels decreased rapidly, with the remaining antibody inactivated, such that tumor exposure to intact RG7356 might have been too short-lived to procure clinical efficacy. Alternatively, patient heterogeneity in CD44s and CD44v expression may explain the observed activity. Four of 9 patients with tumors expressing predominantly CD44s showed some tumor shrinkage (1% to 22% decrease), whereas none was observed in tumors predominantly expressing CD44v. Inhibition of the CD44s-HA interaction may be important for RG7356 clinical activity, as also demonstrated in preclinical studies [4].

We incorporated an exploratory immuno-PET analysis to assess biodistribution of ^89^Zr-RG7356. PET imaging using therapeutic agents as tracers has been developed in preclinical models and can potentially be used in early drug development to confirm expression of drug target in tumor sites and assess whole body biodistribution over time. These findings can help optimize dosage for better tumor targeting and prediction of response and understanding of side effects [9, 10]. Preclinical evaluation previously demonstrated specific uptake of ^89^Zr-RG7356 comparable with that observed in our study [11]. Additionally, we observed significant accumulation of ^89^Zr-RG7356 in the liver, another “sink” organ potentially influencing drug availability. Unlike previous PET-imaging with novel-labeled mAbs to detect residual tumor lesions [12] or predict radio-immunotherapy dose exposure [13], this is one of the first phase I trials to our knowledge incorporating immuno-PET to support early development of a novel compound by describing its biodistribution and tumor targeting at different doses. Tumor targeting of ^89^Zr-RG7356 at doses ≥200 mg suggests that the disappointing activity was unrelated to drug delivery to the tumor. Alternatively, binding of RG7356 alone might not be sufficient to achieve antitumor activity, with preclinical data revealing that only 1 of 2 CD44-positive xenografts respond to RG7356 [11, 14]. Combination regimens may be needed to overcome potential drug resistance or tumor escape that may limit the efficacy of RG7356 as monotherapy.

In conclusion, in a heavily pretreated group of patients with metastatic and/or locally advanced CD44-expressing solid tumors, RG7356 was well tolerated. The MTD was determined at 1,500 mg in the q2w regimen, but not defined in the qw regimen owing to early study termination for reasons unrelated to safety. Clinical efficacy was modest, with a best response of SD. The data support the use of immuno-PET in early clinical development, with proof-of-concept demonstrated using ^89^Zr-RG7356 PET. Tumor targeting from doses ≥200 mg warrants further investigation of RG7356, perhaps in combination regimens.

## MATERIALS AND METHODS

### Patient selection

Detailed inclusion/exclusion criteria are provided in the Supplemental Material (online only). Briefly, patients with histologically confirmed metastatic and/or locally advanced malignant, CD44-expressing solid tumors not amenable to standard therapy were included. During the trial, it became apparent that CD44 isoform expression levels vary significantly among patients. Preclinical evidence also suggests that RG7356 preferentially interferes with binding of the CD44s isoform to HA [4]; thus, CD44s could potentially serve as a biomarker for prediction of response. Therefore, in the qw cohort, patients with tumors that express predominantly CD44s, including breast cancer, melanoma, renal and lung cancer, were preferentially included. All patients gave informed consent prior to any study procedure.

### Study design

This first-in-human, open-label, multicenter, phase I, two-arm, dose-escalation, and imaging study (ClinicalTrials.gov Identifier NCT01358903) was approved by medical ethics committees of 6 participating centers in France, The Netherlands, and the United States and performed in accordance with the Declaration of Helsinki. A safety study management team of participating investigators and the study sponsor met every other week to review the data and agree on dose escalation and risk mitigation plans. Patients in Arm A received RG7356 in an escalating “3 + 3” design from 100 up to 2,250 mg q2w or 675 or 1,350 mg qw. In an exploratory imaging substudy, patients in Arm B received ^89^Zr-labeled RG7356. After Arm B completion, patients continued treatment with the highest Arm A dose that was cleared for DLT at that time point, and were evaluated per the Arm A assessment schedule.

Primary objectives were to describe safety and PK profiles of escalating RG7356 doses and to establish the MTD. DLT and MTD definitions are provided in the Supplemental Material (online only). Secondary objectives comprised describing antitumor activity (standard RECIST criteria, version 1.1) and determining the recommended RG7356 dose schedule for an extension cohort. Exploratory objectives included evaluating PD effects of RG7356 on blood, tumor, and skin samples. Additionally, DCE-MRI and FDG-PET were investigated. PET using ^89^Zr-RG7356 evaluated the *in vivo* biodistribution of ^89^Zr- RG7356.

### Treatment

Arm A starting dose of 100 mg was chosen based on the predicted threshold concentration required for tumor stasis and dose-escalation in increments ≤100% until clinically significant toxicity (two Grade 2 or one Grade ≥3 toxicity, or drug-related toxicity deemed significant by the investigator) was observed, at which time the maximum-allowed increase would not exceed 50%. Doses were increased up to 2,250 mg. Patients were enrolled into 8 dose cohorts to receive RG7356 q2w, and into 2 dose cohorts (675 and 1,350 mg) for the qw regimen (Supplemental Material, online only). Patients continued treatment in the same cohort until disease progression, unacceptable toxicity, or withdrawal. In Arm B, 37 MBq of ^89^Zr-label containing 1 mg of RG7356 was administered as a 20 mL bolus after a predetermined amount of cold RG7356.

### Safety

Patients were monitored for AEs for 2 hours post infusion at each visit. DLTs were assessed over a safety-monitoring period of 28 days and as necessary throughout the study. Routine hematology and biochemistry laboratory assessments were performed before and after treatment on days 1 and 8 of each cycle.

### Pharmacokinetic analysis

Blood sampling was performed on each treatment day before and after infusion, and on days 2, 3, and 8 after the first treatment cycle. Estimation of PK parameters was performed using standard noncompartmental (model independent) methods. Actual sampling time was used to calculate PK parameters. PK analysis was performed using Phoenix^®^ WinNonlin^®^ version 6.2.

### Biomarker analysis

Blood samples were taken during cycles 1, 2, and 4 for immunophenotyping of circulating immune cells and for cytokine analysis (Supplemental Material, online only). To assess a potential alteration of macrophages in the tumor due to RG7356, paired tumor tissue samples collected at baseline and cycle 3 day 1 were analyzed for CD68^+^ macrophages via IHC (details of these procedures can be found in the Supplemental Material, online only). Tumor biopsies were collected to assess CD44 expression at baseline, on day 1 cycle 3, and optionally at tumor progression (Supplemental Material, online only).

### Imaging

To monitor tumor interstitial fluid pressure, DCE-MRI assessments were performed before and 48 ± 12 hours after end of infusion in cycle 1 and up to 12 hours after end of infusion in cycle 3 for doses > 400 mg for patients with at least 1 tumor lesion ≥1 cm outside the thoracic cavity, without metal implants or impaired renal function. Tumor metabolism changes were studied using FDG-PET according to European Association of Nuclear Medicine guidelines [15] before start and before the second and fourth treatment cycles. Whole-body PET with ^89^Zr-labeled RG7356 (Supplemental Material, online only) was performed in a subgroup of patients on days 1, 2, and 5 post injection to evaluate tumor targeting and organ biodistribution.

## SUPPLEMENTARY MATERIAL


