# Supplementary material for: First-in-human phase I clinical trial of RG7356, an anti-CD44 humanized antibody, in patients with advanced, CD44-expressing solid tumors
Source: Oncotarget. 2016 Aug 5;7(48):80046–58. doi: 10.18632/oncotarget.11098 (PMC5346770; doi:10.18632/oncotarget.11098)
Supplement: Supplementary file 1 [file oncotarget-07-80046-s001.pdf]

First-in-human phase I clinical trial of RG7356, an anti-CD44 humanized antibody, in patients with advanced, CD44-expressing solid tumors

Supplementary Material

Materials and Methods

Inclusion/exclusion criteria

Patients were included according to the following specific criteria: histologically confirmed metastatic and/or locally advanced malignant, CD44-expressing solid tumors (tested ≥1+ for CD44 positivity on pretreatment biopsy) not amenable for standard therapy and radiologically measurable or clinically evaluable disease as well as adequate hematological and end organ functions. Exclusion criteria were any other anticancer treatment or major surgery administered concurrently or ≤28 days before start of treatment with RG7356, known central nervous system metastases, active or uncontrolled bleeding, diabetes mellitus, cardiovascular, pulmonary, and/or infectious disease, pregnancy or breastfeeding, immune suppressive or immune therapy, hemolytic anemia, grade >2 neuropathy, recent history of venous or arterial thrombosis, as well as dermatological diseases potentially predisposing to severe skin reactions. All patients gave their informed consent prior to any study procedure.

Treatment with RG7356

RG7356 was supplied as 200-mg vials (Ro 542-9083/F04) or 250-mg vials (Ro 542-9083/F05) by the sponsor (F. Hoffmann-La Roche Ltd, Basel, Switzerland). RG7356 was given as a 2-hour intravenous infusion, with a single cycle constituting 1 or 2 infusions for the biweekly (q2w) and weekly (qw) regimens, respectively.

A preplanned specified risk management plan in case of infusion-related reactions (IRRs) included reduced infusion rate and premedication (acetaminophen 650–1,000 mg

orally, diphenhydramine 25–50 mg intravenous or an alternative antihistamine, and dexamethasone 10 mg intravenous or equivalent dose of another corticosteroid). When the first patient in the q2w cohort developed a grade 2 IRR in response to RG7356 100 mg, standard premedication was instituted before treatment with RG7356 and subsequently from cohort 9 (675 mg qw). Following the incidence of a reversible grade 4 IRR in a separate phase I trial of RG7356 in patients with acute myeloid leukemia (clinicaltrials.gov NCT01641250), the infusion protocol was adapted such that the first infusion was started at a rate of 10 mg/hour for the first 30 minutes, escalating every 30 minutes to a maximum speed of 800 mg/hour. Subsequent infusions could start at 20 mg/hour and escalate at 15 minutes provided no IRR grade 2 or higher event had occurred.

Safety assessments included frequent measurements of blood pressure, heart rate, respiratory frequency, body temperature, and blood chemistry as well as hematology during and after infusions. Urine sampling and an electrocardiogram were performed at regular intervals.

Dose-limiting toxicity/maximum tolerated dose definitions

A dose-limiting toxicity (DLT) was defined as a study drug–related adverse event, graded according to the NCI Common Terminology Criteria for Adverse Events version 4.0, of grade ≥3 with the exception of isolated grade 3 neutropenia <7 days and/or grade 3 thrombocytopenia and an episode of grade ≥3 nausea, vomiting, and/or diarrhea lasting <24 hours after the implementation of adequate treatment. In addition, the failure to recover from toxicity resulting in dose delay >7 days as well as grade ≥3 elevations in aspartate aminotransferase and alanine aminotransferase lasting ≥7 days or worsening by ≥2 grades compared with baseline were considered a DLT. The maximum tolerated dose was defined as

the dose level below the level at which a DLT occurred in either 2 of 3 or 2 of 6 treated patients.

Pharmacokinetic analysis

Total clearance, volume of distribution, half-life, time to peak concentration, peak concentration, area under the curve (AUC), individual and mean serum RG7356 concentrations versus time, and interpatient variability were determined for the pharmacokinetic analysis.

Assessment of circulating monocytes (CD14) (A), cytokines (B), tumoral CD44 expression (C), and tumor-infiltrating macrophages (CD68) (D)

(A) An assessment of circulating monocytes was performed on 423 whole blood specimens, which were shipped ambient in BCT CytoChex blood collection tubes from sites to Covance CLS sites in Geneva, Switzerland and Indianapolis, IN, USA (between June 2011 and December 2013) using the monocytes panel, a 2-tube, 4-color panel. Testing was performed on BD FACSCanto II Flow Cytometer System (BD Biosciences, San Jose, CA, USA) equipped with 405-nm, 488-nm, and 633-nm lasers and a FACSLoader using DIVA Software. The antibodies used were CD14 APC (clone M9), anti-CD16 PE (clone 3G8), anti-CD45 V500 (clone H130), and Anti HLA-DR PerCP (clone L243). Analysis was performed according to CCLS SOP 800690-180, CD14+-Monocytes in Whole Blood (CD14, CD16, HLA-DR) by Flow Cytometry, using DIVA Software on the BD FACSCanto II.

(B) Analysis of cytokines for BP25385 was performed by Myriad RBM multiplex panel (Austin, TX, USA). All samples were stored at −80°C until tested. The samples were thawed at room temperature, vortexed, spun at 4,000 RPM for 5 minutes for clarification, and volume was removed for MAP analysis into a master microtiter plate. Using automated pipetting, an aliquot of each sample was introduced into the capture microsphere multiplex of the Multi-Analyte Profile. The mixture of sample and capture microspheres were thoroughly mixed and incubated at room temperature for 1 hour. Multiplexed cocktails of biotinylated reporter antibodies for the multiplex were then added robotically, and after thorough mixing, were incubated for an additional hour at room temperature. Multiplex was developed using an excess of streptavidin–phycoerythrin solution, which was thoroughly mixed into the multiplex and incubated for 1 hour at room temperature. The volume of each multiplexed reaction was reduced by vacuum filtration and the volume increased by dilution into matrix buffer for analysis. Cytokines were reported by multiplex Core 1. Analysis was performed in a Luminex 100 instrument and the resulting data stream was interpreted using proprietary data analysis software developed at Rules-Based Medicine. For the multiplex, both calibrators and controls were included on each microtiter plate. Eight-point calibrators were run in the first and last column of each plate and 3-level controls were included in duplicate. Testing results were determined first for the high, medium, and low controls for the multiplex to ensure proper assay performance. Unknown values for each of the analytes in the multiplex were determined using 4- and 5-parameter, weighted and nonweighted curve-fitting algorithms included in the data analysis package.

(C) Immunohistochemical analysis of tumoral CD44 expression was performed by Ventana Medical Systems Inc. (VMSI, Tucson, AZ, USA). In total, 133 formalin-fixed paraffin-embedded tumor tissue specimens (102 pretreatment and 31 on-treatment tumor

biopsies) from 6 clinical centers participating in BP25385 were received at VMSI. Hematoxylin and Eosin stains were performed, and an assessment of tissue quality as well as tumor content was reviewed by the pathologist. CD44 immunohistochemical analysis was carried out at VMSI's CAP/CLIA laboratory on Benchmark XT using clone SP37 (VMSI, Catalog No. 790-4537) as the primary antibody and was run according to package insert conditions. An immunoglobulin matched rabbit monoclonal immunoglobulin G antibody (VMSI, Catalog No. 760-1029) was used as a negative-control antibody to examine the presence of secondary antibody and detection chemistry reactivity.

(D) Immunohistochemical analysis of tumor-infiltrating macrophages was performed by HistogeneX (Antwerp, Belgium). Formalin-fixed paraffin-embedded slides from tumor samples were received via VMSI at HistogeneX between December 2011 and March 2014. CD68 immunohistochemical analysis was performed on Ventana Benchmark XT (Ventana Medical Systems, Inc., Tucson, AZ, USA) using clone KP-1 as the primary antibody. Immunohistochemical analysis was performed according to HistogeneX staining SOPs TE.HP.34 (CD68), as well as HistogeneX scoring SOP DM.IQ.7.

Imaging

DCE-MRI (to monitor tumor interstitial fluid pressure, and FDG-PET (to assess changes in tumor metabolism) results were analyzed at a central core laboratory (IXICO, London, UK).

Immuno-PET ^{89}Zr- RG7356 imaging protocol

Radiolabeling of RG7356 with ^{89}Zr was performed as described according to state-of-the-art Good Manufacturing Practice standards (1). Patients received 1-mg 37 MBq ^{89}Zr-

labeled RG7356 as a bolus of 20 mL followed by 10 mL physiologic saline flushing within 2 hours after administration of the first dose of unlabeled RG7356. Doses of unlabeled RG7356 ranged from 0 mg (1 patient) to 99 mg (3 patients), 199 mg (2 patients), 449 mg (5 patients), and 674 mg (2 patients).

Within 2 hours of administration of ^{89}Zr-labeled RG7356, 3 whole-body positron emission tomography (PET) scans per patient were performed at 2 hours, 1 day, and 5 days post injection. Acquisition protocol: at each PET scan, ^{89}Zr-PK samples were taken. PET/computed tomography (CT) scans were performed on Philips scanner (Gemini series). PET data were normalized, corrected for decay, randoms, dead time, scatter, and attenuation, and reconstructed using a time-of-flight list-mode ordered-subsets expectation maximization reconstruction method with a matrix size of 144 × 144 and a voxel size of 4 × 4 × 4 mm^3. In addition, for each time point, a 50-mA low-dose CT scan was acquired for attenuation-correction purposes. Corresponding CT images were reconstructed with an image matrix size of 512 × 512 and a voxel size of 1.17 × 1.17 × 5 mm^3.

Acquired images were analyzed by visual inspection and measurements of standardized uptake value (mean, peak, and max; corrected for body weight) and percentage of injected dose with respect to organs and tumor lesions.

Results

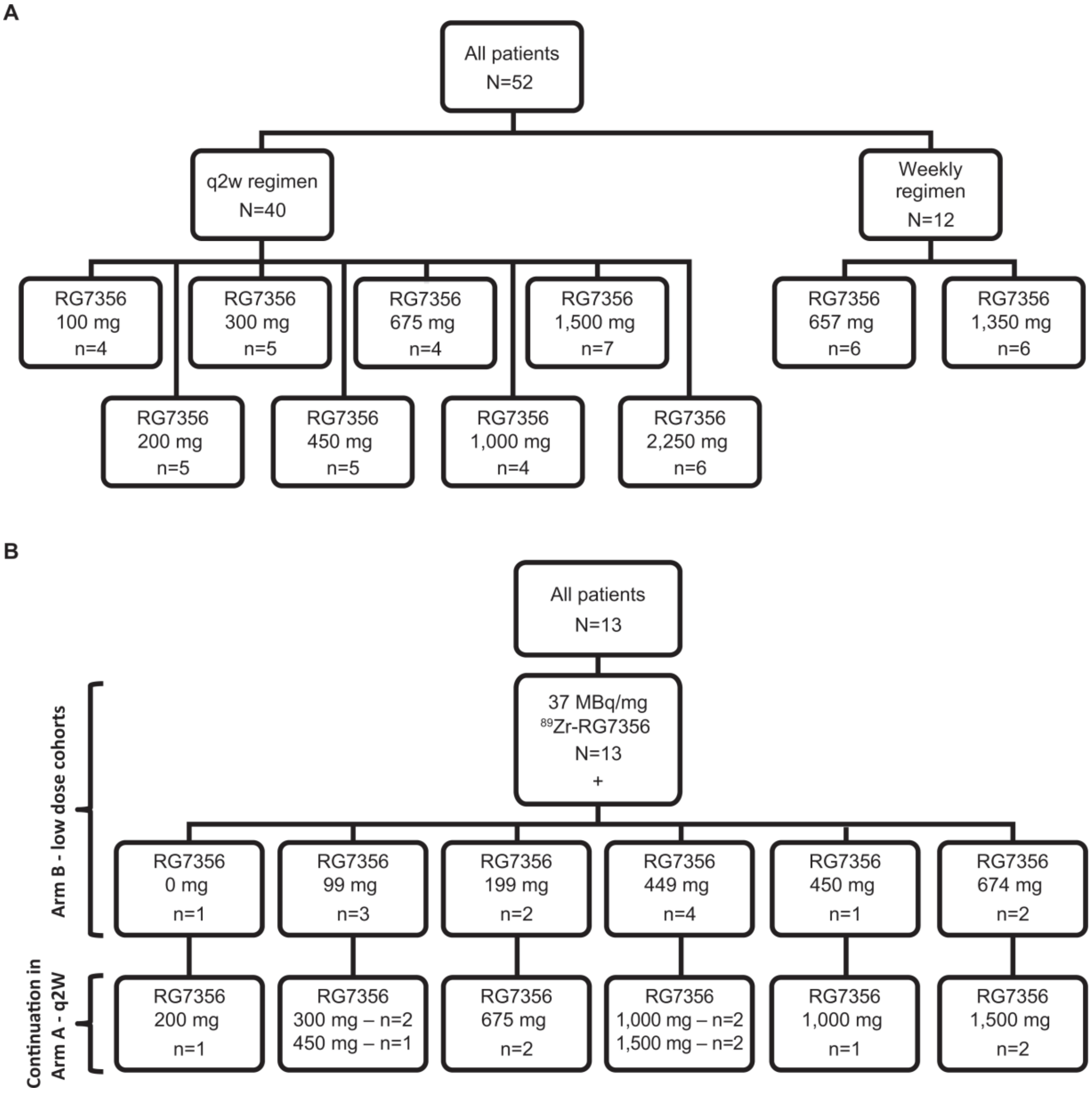

Supplementary Figure 1. Summary of patient disposition in Arm A (A) and Arm B (B)

Biomarker analysis

Two patients experienced dose-limiting headache. Overall, cytokine levels in 2 patients who experienced dose-limiting headache were similar to those in other patients treated with

RG7356. The first patient (Patient 3021 treated with the qw regimen) did not undergo sampling for cytokine analyses at the time when the dose-limiting event of headache occurred (study day 8). The second patient (Patient 1008 treated with the q2w regimen) underwent cytokine sampling on day 3, while the dose-limiting headache occurred on day 4. Cytokine levels on day 3 were similar in Patient 1008 compared with those in other patients treated with RG7356.

Reference

1. Verbruggen A, Coenen HH, Deverre JR, Guilloteau D, Langstrom B, Salvadori PA, et al. Guideline to regulations for radiopharmaceuticals in early phase clinical trials in the EU. Eur J Nucl Med Mol Imaging. 2008; 35: 2144-2151. doi: 10.1007/s00259-008-0853-7
